# Impact of titanium nanoparticles on germination and early growth of faba bean (*Vicia faba* L.)

**DOI:** 10.1038/s41598-025-18071-1

**Published:** 2025-09-12

**Authors:** Somaia Youssif Abdelmagid, Fatma Abd El Lateef Gharib, Eman Zakaria Ahmed

**Affiliations:** Botany and Microbiology Department, Faculty of Science, Helwan University, Helwan, Egypt

**Keywords:** Faba bean (*Vicia Faba* L.), Titanium dioxide nanoparticles, Germination, Seedling growth, Enzymatic activity., Biochemistry, Biological techniques, Biotechnology, Plant sciences

## Abstract

This study explores the potential of biosynthesized titanium dioxide nanoparticles (TiO_2_NPs), capped with phytochemicals from *Zygophyllum simplex* extract, in enhancing seed germination and related indices of faba bean (*Vicia faba* L.), aiming to support sustainable and biocompatible approaches in nano-agriculture. Specifically, the study assessed the impact of biogenic TiO_2_NPs (average size ≈ 12.8 nm) on seed germination, early plant growth parameters such as root and shoot length, and germination percentage, as well as the activity of selected antioxidant and hydrolytic enzymes in faba bean. Seeds were soaked for 6 h in an aerated solution containing six concentrations of TiO_2_NPs (1, 5, 10, 25, 50, and 100 µM), alongside a control group. Germination was conducted in dark at 25 ± 0.5 °C for six days. The results demonstrated that TiO_2_NPs at concentrations up to 25 µM significantly enhanced germination percentage, seedling growth parameters, and increased the activities of antioxidant enzymes; catalase (CAT), peroxidase (POD), and superoxide dismutase (SOD), as well as α-amylase and protease enzymes, total soluble sugar and protein content, in comparison with non-treated seeds. However, TiO_2_NPs at a concentration of 50, and 100 µM significantly reduced germination rates, inhibited seedling growth, and decreased enzyme activities relative to the control. These findings suggest that nano-titanium, particularly at a concentration of 10 µM, effectively enhances germination potential, subsequent seedling growth, and enzyme activity in Faba beans. Further research on the impact of these nanoparticles on vegetative growth and yield could provide valuable insights based on the positive effects observed during germination and early seedling development.

## Introduction

Legume crops are a sustainable and widely cultivated source of high-protein food. Among them, faba bean (*Vicia faba* L.) is a historically significant and globally important crop^1^. Major producers include Mediterranean countries, Egypt, Ethiopia, China, India, and parts of Europe and Northern Africa^2^. As a multipurpose crop, faba bean (Fabaceae family) plays a valuable role in food security, animal feed, and ecological sustainability through nitrogen fixation, reducing the need for synthetic fertilizers^3^.

In recent years, nanotechnology has emerged as a promising tool in agriculture, offering innovative solutions to enhance crop productivity and resilience. Nanoparticles may be applied in agriculture through several means such as seed priming, soil, and foliar application^4^. Nanoparticles synthesized from green synthesis approach produce more catalytic activity and limit the use of expensive and toxic chemicals^5^. Metal oxide NPs are increasingly recognized as valuable technological tools in addressing current and future obstacles related to crop productivity^6^. Amongst the metal oxide nanoparticles, TiO_2_ nanoparticles are more remarkable nanomaterial due to its photocatalytic properties, chemical stability, non-toxicity, and their ability to improve plant growth and physiological performance^7^. It has enormous uses in cosmetic industry, pollution control and antibacterial coatings^8^. In mung bean (*Vigna radiata*), foliar application of TiO_2_NPs has been shown to significantly enhance shoot and root length, root area, nodule formation, chlorophyll content, and total soluble leaf protein^9^. Furthermore, Mathew et al.^10^ reported that TiO_2_NPs synthesized using *Cuminum cyminum* extract effectively promoted seed germination and stimulated various growth parameters in mung bean. The synthesis of TiO_2_ nanoparticles using plant-based methods has been widely explored due to their eco-friendly and cost-effective nature^8^. In addition, the use of *Zygophyllum simplex* L. leaf extract for the biosynthesis of silver nanoparticles has been demonstrated, further supporting the potential of plant-mediated synthesis routes in nanotechnology^11^.

Faba bean (*Vicia faba* L., var. ‘Nubaria 4’) seeds are of significant agricultural importance. However, the tough seed coat of faba beans impedes water absorption, thereby delaying germination. Recent research has explored the agricultural application of titanium dioxide nanoparticles (TiO_2_NPs) to improve seed germination and seedling emergence. Titanium nanoparticles enhance seed hydration by modifying the surface properties of seeds, increasing their hydrophilicity, which in turn promotes faster and more efficient water absorption. Consequently, understanding the effects of TiO_2_NPs on dormancy-breaking, germination promotion, and potential morphological changes in faba bean seedlings is essential. This knowledge will help to evaluate the benefits or harms posed by TiO_2_NPs, ultimately enabling the development of strategies to ensure sustainable production and food safety for consumers.

To the best of our knowledge, the application of TiO_2_ nanoparticles synthesized from plant extracts in germination studies of higher plants is still limited. Currently, there are no reports on the green synthesis of TiO_2_ nanoparticles using *Zygophyllum simplex* extract. This study is one of the first to utilize green-synthesized TiO_2_ nanoparticles from *Z. simplex* in the seed germination of faba beans (*Vicia faba* L.), potentially paving the way for future research on the use of green-synthesized TiO_2_ nanoparticles in germination studies.

This study aimed to assess the response of Faba bean (*Vicia faba*) plants to soaking in various concentrations of green-synthesized TiO_2_ nanoparticles (TiO_2_NPs) by examining their effects on germination percentage, seedling growth, and enzymatic activity in comparison to untreated control seeds. The goal was to identify the most effective TiO_2_NPs concentrations for potential agricultural applications.

## Materials

The Horticulture Research Institute, Agriculture Research Center, Ministry of Agriculture, Giza, Egypt provided a consistent batch of variety Nubaria 4 of faba beans (*Vicia faba* L.) seeds. *Zygophyllum simplex* plant was collected in March 2024 from Wadi Degla Protectorate in Cairo, Egypt, identified by Dr. Eman Zakaria assistant Professor at Faculty of Science – Helwan University. A voucher plant specimen was kept at the herbarium of Helwan University’s Faculty of Science in Egypt with voucher number 0111033.

### Synthesis, and characterization of titanium dioxide nanoparticles

Titanium dioxide nanoparticles (TiO_2_NPs) were synthesized using titanium isopropoxide (Ti[OCH(CH₃)_2_]_4_, 97%) and *Zygophyllum simplex* extract. The synthesis and characterization techniques, including measurements of size, phase composition, and surface charge, confirmed the successful formation and stability of the TiO_2_ nanoparticles.

### Plant materials and treatments

A homogenous lot of faba beans seeds variety (Nubaria 4) was surface sterilized using a 2.5% sodium hypochlorite solution for 3 min and rinsed with distilled water. The seeds were then divided into 7 groups, each of 100 seeds. These groups were soaked in different solutions. The soaking process involved placing fixed number of seeds from each group into glass containers containing a consistent volume (100 ml) of the treatment solutions Titanium Nanoparticles (TiO_2_NPs ≈ 12.8 nm) at concentrations of 0.0, 1, 5, 10, 25, 50 and 100 µM, distilled water was used for control treatment, and allowing them to soak for 6 h at a temperature of 25 °C. Following this, the seeds of the control and each treatment were washed thoroughly with distilled water, then transferred to germination pots (20 cm long, 15 cm wide, and 10 cm depth), containing sterilized sandy soil for 6 days. The experiment involved the replication of each treatment five times. A grand total of 35 germination plastic pots, each consisting of 20 seeds, were utilized. Additionally, all germination pots were carefully positioned within a growth chamber at 65% relative humidity, and a temperature of 25 °C ± 0.5, with alternating periods of darkness and light. These conditions were maintained consistently to enable controlled experimentation.

### Growth measurement

#### Germination indices

To evaluate the effect of titanium nanoparticles treatments, germination percentage and seedling vigor indices were calculated as below.

The germination percentage (GP) was calculated as a percentage of total germinated seeds 6 days after soaking (DAS) for each treatment, according to the following:

$$\:\text{G}\text{e}\text{r}\text{m}\text{i}\text{n}\text{a}\text{t}\text{i}\text{o}\text{n}\:\text{p}\text{e}\text{r}\text{c}\text{e}\text{n}\text{t}\text{a}\text{g}\text{e}\:\left(\text{G}\text{P}\right)=\:\frac{n}{N}\times\:100$$

where n is number of germinated seeds, and N is total number of seeds for bioassay.

The seedling vigor index (SVI) was measured according to Noorhossein et al.^12^ at 6 DAS, using the following formula:

$$\:\text{S}\text{V}\text{I}\:\:=\text{l}\text{e}\text{n}\text{g}\text{t}\text{h}\:\text{o}\text{f}\:\text{s}\text{e}\text{e}\text{d}\text{l}\text{i}\text{n}\text{g}\:\times\:\text{G}\text{P}$$

#### Growth measurement

To evaluate various growth parameters of Faba bean, including plumule and radicle length (cm), as well as fresh and dry weights (g) per seedling, 10 randomly selected 7-day-old seedlings were taken from each treatment and the control group. The collected samples were either dried in an oven at 70 °C for 24 h until a constant dry weight was achieved, and used to measure the levels of total soluble sugars and total soluble proteins, or fresh samples were used to determine fresh weight and conduct assays for antioxidant enzymes as well as α-amylase and protease activities in TiO_2_NPs -treated seedlings.

#### Hydrolytic enzyme activity

Activiti of α-amylase, and protease were assessed in 7-day-old seedlings of Faba bean (Nubaria 4 variety) that were presoaked for 6 h in TiO_2_NPs solutions at concentrations of 1, 5, 10, 25, 50, and 100 µM, with distilled water used as the control.

### Sample extraction

Two grams of fresh faba bean seedlings were ground in a pre-chilled mortar and pestle with 10 ml double distilled water till a paste was formed, and the extracts were centrifuged at 4000 rpm and 4° C for 20 min. The obtained clear supernatants solutions were used for various enzymatic assays.

### α-amylase enzyme (EC 3. 2.11)

The α-amylase activity in Faba bean was assayed following the method described by Bergmeyer^13^. The assay mixture included 0.5 ml of starch in phosphate buffer (pH 7.0), 0.5 ml of double distilled water, and 0.5 ml of enzyme extract. After incubating the mixture at 25 °C for 10 min, 1 ml of a color reagent (1% dinitro salicylic acid) was added, followed by boiling the mixture in a water bath for 10 min. The mixture was then cooled in an ice bath, diluted to 10 ml with distilled water, and the absorbance was measured at 546 nm using a Cecil CE 1010 spectrophotometer.

### Protease enzyme (EC 3.4.21.40)

Protease activity was determined using the method described by Bergmeyer^13^. The reaction mixture consisted of 1 ml of casein (1%) in phosphate buffer (pH 7.5) and 1 ml of enzyme extract. After incubating for 1 h at 37 °C, 2 ml of 10% trichloroacetic acid was added to stop the reaction. The mixture was then centrifuged at 4000 rpm for 20 min at 4 °C. The amino acid content in the supernatant was measured following the procedure by Lowry et al.^14^ to determine protease activity based on the amount of amino acid produced from casein hydrolysis. Specifically, 1 ml of Faba bean extract was mixed with 5 ml of a freshly prepared solution (50:1 v/v) of 2% sodium carbonate in 4% sodium hydroxide and 0.5% copper sulfate in 1% sodium tartrate. After allowing the mixture to stand for 10 min, 0.5 ml of Folin reagent was added, and the final volume was adjusted. The optical density was measured spectrophotometrically at 750 nm after 30 min.

### Antioxidants enzymes

#### Catalase (EC 1.11.1.6)

The seedling of faba beans was employed to generate enzyme extract as shown by Kar and Mishra^15^. 0.5 g of fresh seedling was grinding within 10 mL chilled phosphate buffer (with a concentration of 0.1 M Na/K phosphate, set at pH 6.8). The mixture underwent centrifugation for 10 min at 6000 rpm and 4 °C. The resultant supernatant was then diluted to a predetermined volume and utilized for the assessment of catalase (CAT) activity.

The procedure for measuring CAT activity was adapted from the method introduced by Góth^16^. This involved introducing 1 ml of H_2_O_2_; (specifically, a solution containing 65 mM H_2_O_2_ in N/KP at pH 7.4) and 0.2 ml of the crude enzyme extract into the reaction vessel. The reaction was permitted to proceed at a temperature of 25 °C for 4 min. To terminate the reaction, 1 ml of ammonium molybdate (concentrated at 4 g L^− 1^) was added. The residual H_2_O_2_ level was quantified by observing the reduction in absorbance at a wavelength of 405 nm. For comparative, a control experiment was concurrently executed, wherein the enzymatic activity was instantaneously ceased at zero time. The catalase activity was quantified in terms of micromoles of H_2_O_2_ decomposed per microgram of fresh weight per hour.

#### Peroxidase (EC 1.11.1.7)

Peroxidase was evaluated in faba bean seedling as described by Yamane et al.^17^ modified version. The assay mixture consisted of 2.2 mL of 0.1 M K_3_PO_4_ buffer (pH 6.0), 0.5 mL of guaiacol (0.018 mM), 0.2 mL of 30% H_2_O_2_, and 0.1 mL of enzyme extract. The reaction’s color intensity was monitored by measuring absorbance at 436 nm, with readings taken every 30 s for a duration of 3 min. Enzyme activity was quantified and expressed as optical density per microgram of fresh weight per hour.

#### Superoxide dismutase (EC 1.15.1.1)

The activity of superoxide dismutase (SOD) enzyme was determined within fresh faba bean seedlings, adhering to the protocol established by Nishikimi et al.^18^. The assay was facilitated using a commercially available SOD Biodiagnostic Ready Kit from Biodiagnostic Co. The procedure began with the homogenization of 0.25 g of fresh tissue in 5 mL of cold potassium buffer (pH 7.0) with a concentration of 100 mM. This mixture was subjected to centrifugation at 4000 rpm and 4 °C for 15 min. Following centrifugation, 0.5 mL of an absolute cold ethanol/chloroform mixture (in a 60/40, v/v ratio) was combined with 1 mL of the supernatant in a glass tube. The contents were thoroughly mixed for at least 30 s, after which the mixture underwent another round of centrifugation under identical conditions. The supernatant obtained from this extraction process was then utilized in the subsequent assay. To assemble the assay mixture, 2 mL of a 50 mM K_3_PO_4_ buffer adjusted to a pH of 8.5, 1 mM nitrobluetetrazolium, and 1 mM NADH were mixed together in a 10:1:1 ratio. Subsequently, 0.2 mL of the enzyme extract and 0.2 mL of distilled water were added to the mixture. A control assay was also prepared using the same mixture composition, excluding the enzyme extract. This control mixture placed into a clean quartz cuvette. Initiation of the reaction involved the addition of 0.2 mL of 0.1 mM phenazine methosulphate (PMS), which had been diluted 1000-fold prior to use. The cuvette was then carefully inserted into a spectrophotometer maintained at a constant temperature of 25 °C for 5 min. During this time, the spectrophotometer recorded the increase in absorbance at a wavelength of 560 nm for both the control and the seedling sample. The activity of the SOD enzyme was quantified and reported as units per gram of fresh tissue (U g^− 1^).

### Total soluble sugars and total soluble proteins

#### Extraction

A weight of 0.1 g of faba bean seedling was ground in 5 ml ethanol 70%. After centrifugation at 6000 rpm for 15 min, the supernatant was completed to 15 ml by distilled water.

#### Total soluble sugars

Total soluble sugar (TSS) was determined as described by Umbreit et al.^19^ using anthrone technique. 6 ml anthrone solution (2 g l^− 1^ H_2_SO_4_ 95%) was added to 3 ml sample and maintained on a boiling water-bath for 3 min. After cooling, the developed color was measured spectrophotometrically at 620 nm.

#### Total soluble proteins

The procedure of Lowry et al.^14^ was followed. Briefly, 1 ml faba bean extract was mixed with 5 ml freshly mixed solution (50:1 v/v) of 2% sodium carbonate in 4% sodium hydroxide and 0.5% copper sulphate in 1% sodium tartrate. The mixture stood 10 min before addition of 0.5 ml Folin and made up to a definite volume. The optical density of the mixture was measured spectrophotometrically at 750 nm after 30 min.

### Statistical analysis

The dataset was presented as the mean of five replicate measurements, with each measurement involving two seedlings to assess growth parameters, including germination indices and seedling growth. Enzyme activity, total soluble sugars, and total soluble proteins were determined as the average of three replicate measurements. Statistical analysis was conducted using one-way analysis of variance (ANOVA), followed by Duncan’s Multiple Comparison Test, performed with IBM SPSS Statistics for Windows, Version 21. Statistical significance was considered at *P* > 0.05, with means compared using the least significant difference (LSD) at the 5% level.

## Results

**Fig. 1:**
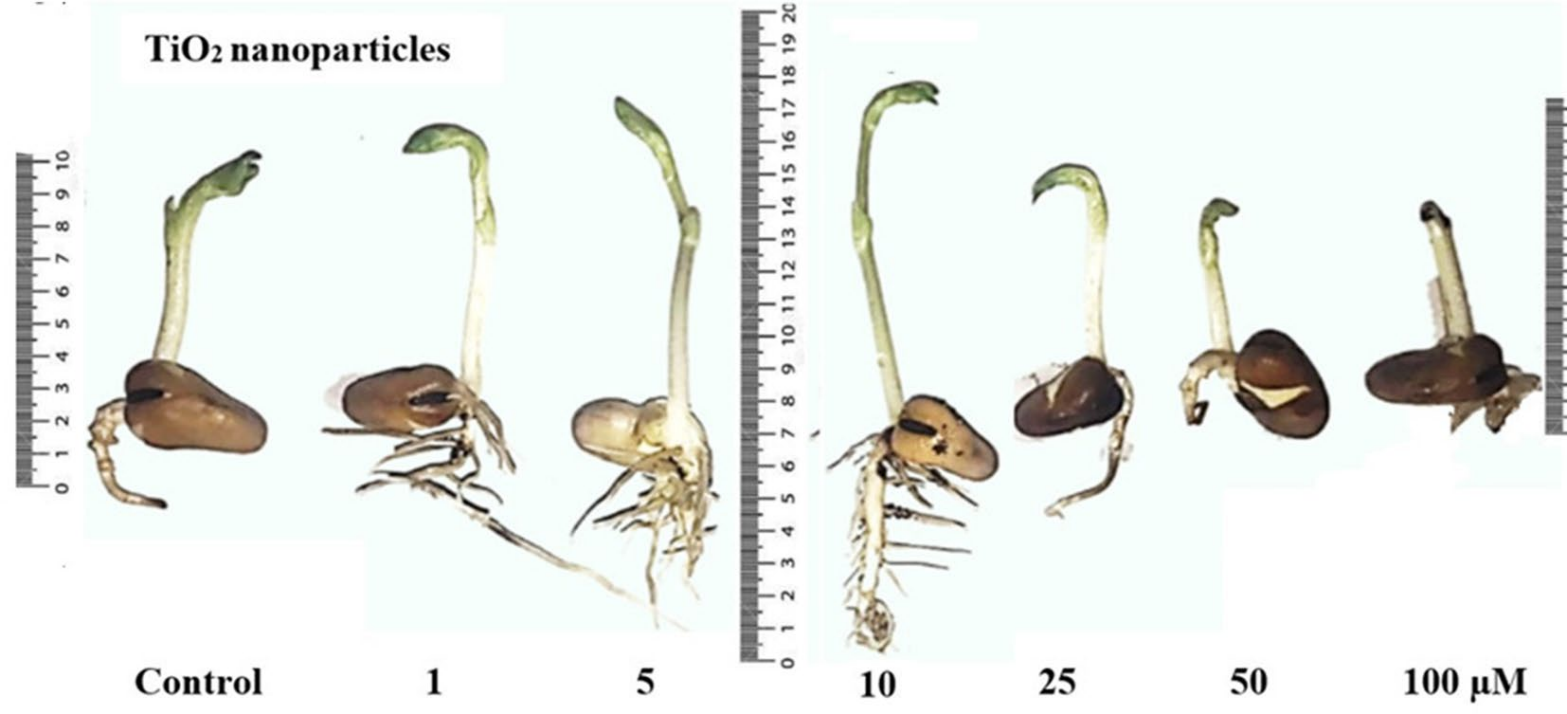
Growth of faba bean (*Vicia faba* L.) seedlings following seed soaking in biogenic titanium dioxide nanoparticles (TiO_2_NPs ≈ 12.8 nm) at concentrations of 0.0, 1, 5, 10, 25, 50, and 100 µM. Seeds were germinated in the dark at 25 ± 0.5 °C for six days.

**Fig. 2:**
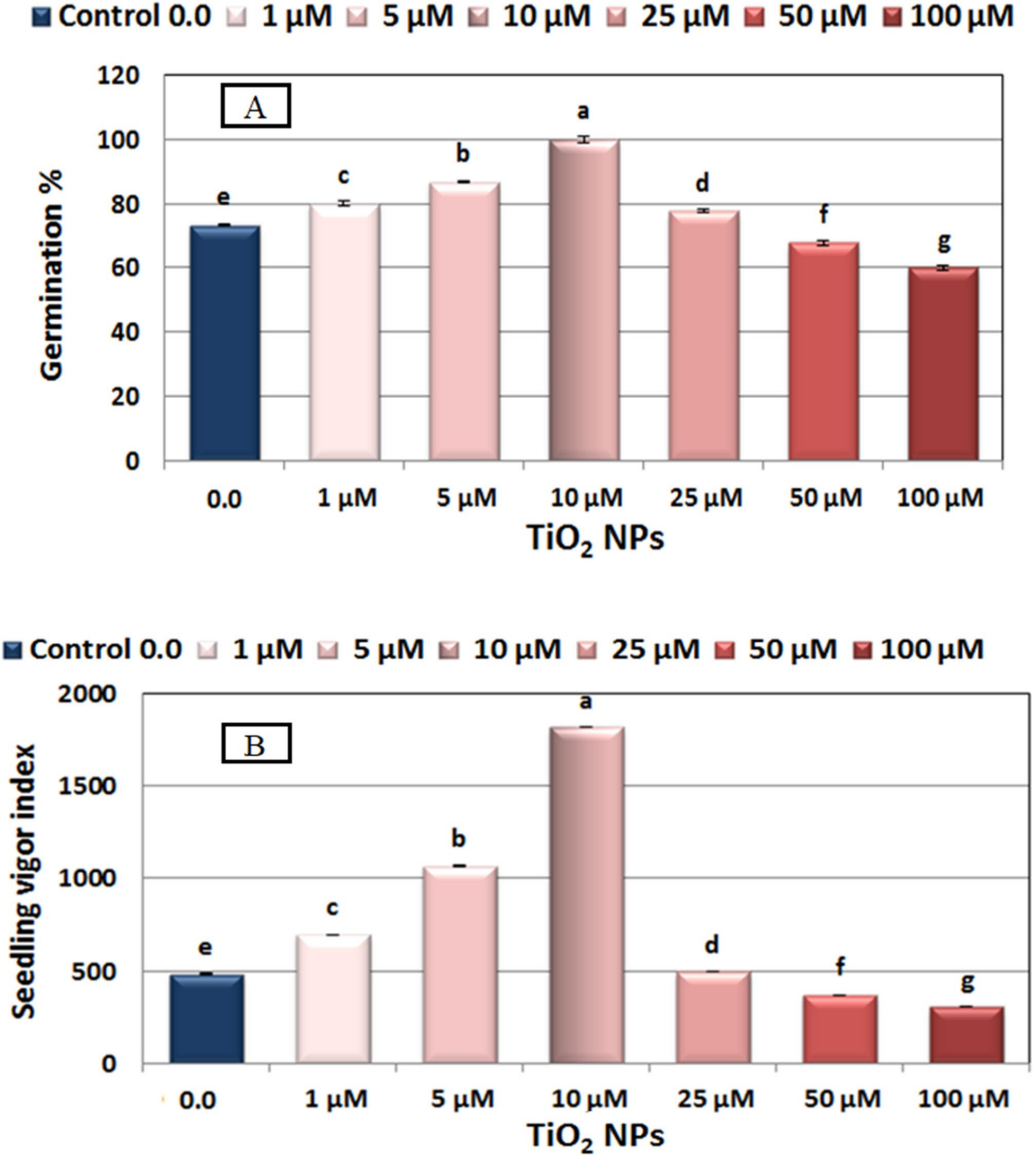
(**A**) Changes in germination percentage, and (**B**) seedling vigor index (SVI) of 7 day- old seedlings of Nubaria 4 variety of faba bean (*Vicia faba* L.) as affected by seed presoaking for 6 h in different concentrations of nano titanium (TiO_2_NPs ≈ 12.8 nm) solution at 0.0, 1, 5, 10, 25, 50 and 100 µM and grown at 25 °C ± 0.5. Each value represents the mean of 5 replicates (each of 10 seeds). Different letters indicate significant differences between treatments (Duncan test *p* ≤ 0.05). Vertical bars represent ± SE.

**Fig. 3:**
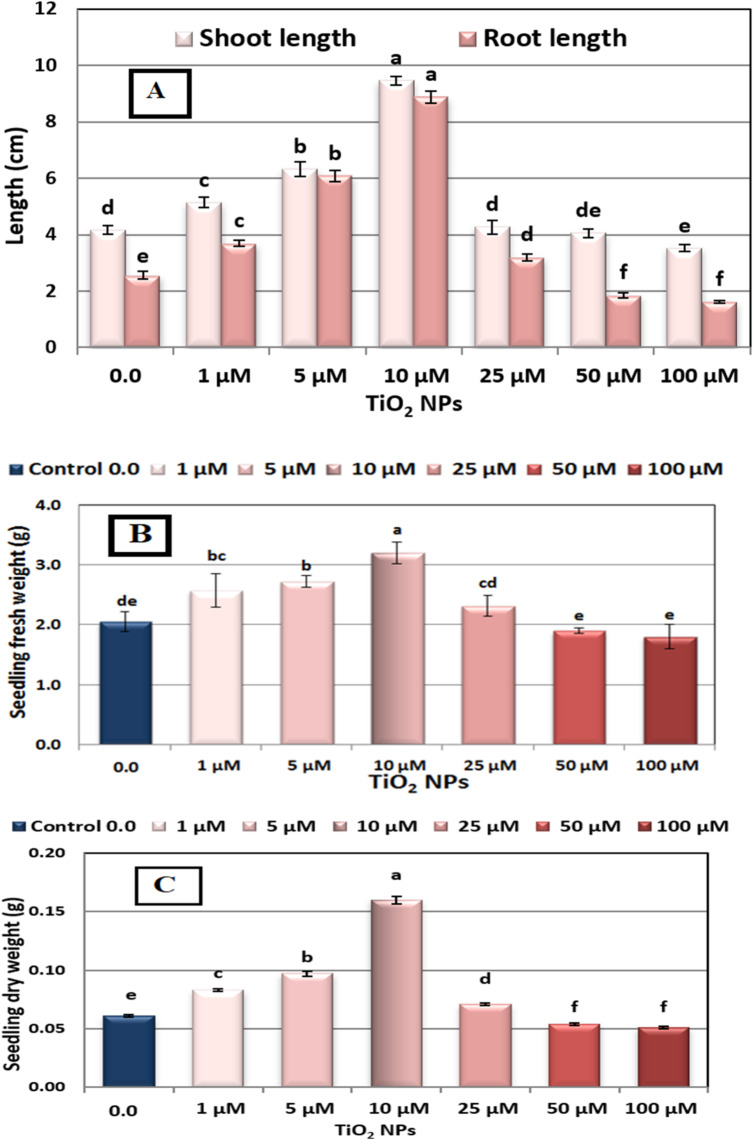
(**A**–**C**). Growth criteria of 7 day-old seedlings of Nubaria 4 variety of faba bean (*Vicia faba* L.) as affected by seed presoaking for 6 h in different concentrations of nano titanium (TiO_2_NPs ≈ 12.8 nm) solution at 0.0, 1, 5, 10, 25, 50 and 100 µM and grown at 25 °C ± 0.5. Each value represents the mean of 3 replicates. Different letters indicate significant differences between treatments (Duncan test *p* ≤ 0.05). Vertical bars represent ± SE.

An experiment was conducted to evaluate the effects of TiO_2_NPs at 1–100 µM concentrations on seed germination percentage, seedling vigor index, and seedling growth of faba bean (Figs. 1, 2 and 3).

### Germination percentage

The data depicted in Fig. 2A illustrate the germination percentage of both control (H_2_O) and TiO_2_NPs treated faba bean. Results demonstrate that soaking the seeds for 6 h in various concentrations of TiO_2_NPs (from 1 µM up to 25 µM) significantly boosted germination percentage of 7-day-old faba bean seedling compared to the control. However, germination percentage gradually declined at higher concentrations. Notably, the most favorable GP performance (100%) was observed when seeds were soaked in solutions containing 10 µM of TiO_2_NPs. in comparison with 73.3% for the control.

Conversely, the germination power was negatively impacted at higher concentrations of TiO_2_NPs (50, and 100 µM), recording − 7.64, and − 18.14%, decline, respectively in comparison to the control treatment (Fig. 2A).

### Seedling Vigor index

The data presented in Fig. 2B demonstrate that the vigor of seedlings, as measured by the seedling vigor index (SVI), was evaluated after soaking faba bean seeds for 6 h in different concentrations (1 µM − 100 µM) of TiO_2_NPs. The results indicate that TiO_2_NPs treatments up to 25 µM had a positive impact on seedling vigor index (SVI) values, consistent with the calculated germination percentages.

Among these treatments, the 10 µM concentration followed by 5 µM, resulted in the most significant increase in SVI, with value approximately 276.20%, and 120.43%, respectively higher than the controls. However, SVI gradually declined at higher concentrations of TiO_2_NPs (50, and 100 µM). Specifically, for the 100 µM concentration, induced the largest decrease in SVI − 36.75% lower than the controls), followed by 50 µM (-18.84% lower than the controls) (Fig. 2B).

### Seedling growth

The presented in Fig. 3A, B, and C demonstrate that the lengths of shoot and root, seedling fresh and dry weights in faba bean were significantly enhanced when soaked in TiO_2_NPs up to 25 µM concentration, and then these values were progressively decreased at higher concentrations compared to the corresponding control seedling. The best results were observed when seeds were soaked in a solution containing 10 µM of TiO_2_NPs, followed by 5µM.

The highest increases in shoot length, root length, seedling fresh weight, and seedling dry weight were observed with TiO_2_NPs at a concentration of 10 µM. These increases were 129.27%, 252.00%, 56.10%, and 162.30% respectively, compared to the control.

On the other hand, TiO_2_NPs at a concentration of 50, and 100 µM were found to slightly reduce shoot length by -2.44%, and, -14.63%, respectively in comparison with their respective controls. Also decreased root length by -28.00%, and − 36.00 and had a negative impact on seedling fresh weight, with reductions of -7.32%, and − 12.20%. Similarly, seedling dry weights were negatively affected by -11.48%, and − 16.39% respectively at 50, and 100 µM level (Fig. 3A, B, and C).

### Activities of α-amylase and protease enzymes

The data in Table 1 indicates that soaking faba bean seeds in TiO_2_NPs solutions up to a concentration of 25 µM enhanced the activities of α-amylase and protease enzymes in 7-day-old seedlings compared to the control. However, enzyme activities progressively declined at higher concentrations. The highest enzyme activities were observed at a 10 µM concentration, with values of 43.60, and 7.56 µg g^− 1^ FW sec^− 1^, followed by 5 µM (39.36, and 6.44 µg g^− 1^ FW sec^− 1^) for α-amylase and protease, respectively. In contrast, control seedlings exhibited enzyme activities of (35.40, and 3.59 µg g^− 1^ FW sec^− 1^ for α-amylase and protease, respectively.

**Table 1:** Changes in enzymatic activities of α-amylase, and protease (µg g^− 1^ FW sec^− 1^), catalase, and peroxidase (µg^− 1^ F W h^− 1^), and superoxide dismutase (U g^− 1^) of 7 day-old seedlings of Nbaria 4 variety of Faba bean ((*Vicia Faba* L.) as affected by seed presoaking for 6 h in different concentrations of nano titanium (TiO_2_NPs ≈ 12.8 nm) solution at 0.0, 1, 5, 10, 25, 50 and 100 µM and grown at 25 °C ± 0.5.

Treatments (µM)	α-amylase	Protease	Catalase	Peroxidase	Superoxide dismutase
	µg g^− 1^ FW sec^− 1^	µg g^− 1^ FW sec^− 1^	µg^− 1^ F W h^− 1^	µg^− 1^ F W h^− 1^	U g^− 1^
Control (H_2_O)	35.40 ^de^ ± 0.35	3.59 ^c^ ± 0.28	4.20 ^f^ ± 0.50	2.60 ^f^ ± 0.40	3.10 ^f^ ± 0.50
TiO_2_ NPs 1 µM	36.90 ^c^ ± 0.32	6.22 ^b^ ± 0.27	4.85 ^d^ ± 0.40	3.10 ^d^ ± 0.30	3.65 ^d^ ± 0.40
TiO_2_ NPs 5 µM	39.36 ^b^ ± 0.33	6.44 ^b^ ± 0.31	5.60 ^b^ ± 0.40	3.75 ^b^ ± 0.40	4.25 ^b^ ± 0.60
TiO_2_ NPs 10 µM	43.60 ^a^ ± 0.24	7.56 ^a^ ± 0.25	6.10 ^a^ ± 0.50	4.14 ^a^ ± 0.50	4.65 ^a^ ± 0.60
TiO_2_ NPs 25 µM	36.16 ^cd^ ± 0.31	6.04 ^b^ ± 0.18	5.10 ^c^ ± 0.60	3.40 ^c^ ± 0.30	4.05 ^c^ ± 0.50
TiO_2_ NPs 50 µM	34.55 ^e^ ± 0.30	2.04 ^d^ ± 0.18	4.40 ^e^ ± 0.70	2.90 ^e^ ± 0.25	3.45 ^e^ ± 0.40
TiO_2_ NPs 100µM	30.08 ^f^ ± 0.29	1.27 ^e^ ± 0.14	3.60 ^g^ ± 0.60	2.40 ^g^ ± 0.30	2.90 ^g^ ± 0.30
LSD at 0.05	0.89	0.69	0.12	0.10	0.12

Each value represents the mean of 3 replicates ± SE SD. Different letters indicate significant differences between treatments (Duncan test, *P* ≤ 0.05).

Conversely, the lowest activity of α-amylase (34.55, and 30.08 µg^− 1^ g^− 1^ FW Sec^− 1^) and protease enzymes (2.04, 1.27 µg^− 1^ g^− 1^ FW Sec^− 1^) were achieved at concentration of 50, and100 µM TiO_2_NPs, respectively (Table 1).

### Activity of antioxidant enzymes

Seed soaking with different concentrations of TiO_2_ nanoparticles (1–100 µM) significantly affected the activities of catalase (CAT), peroxidase (POD), and superoxide dismutase (SOD) in 7-day-old faba bean seedlings (Table 1).

All three antioxidant enzymes showed a similar trend in response to increasing TiO_2_NP concentrations. Activities gradually increased from the control up to 10 µM, where the maximum values were recorded. Catalase activity increased from 4.20 µg^−1^ FW h^−1^ in the control to a peak of 6.10 µg^−1^ FW h^−1^ at 10 µM. Similarly, peroxidase activity rose from 2.60 µg^−1^ FW h^−1^ to 4.14 µg^−1^ FW h^−1^, and SOD activity increased from 3.10 U g^−1^ to 4.65 U g^−1^ over the same concentration range. Beyond 10 µM, enzyme activities declined progressively with increasing concentrations of TiO_2_NPs. At 100 µM, catalase, peroxidase, and SOD activities dropped to 3.60 µg^−1^ FW h^−1^, 2.40 µg^−1^ FW h^−1^, and 2.90 U g^−1^, respectively (Table 1).

### Total soluble sugars and total soluble proteins

**Fig. 4:**
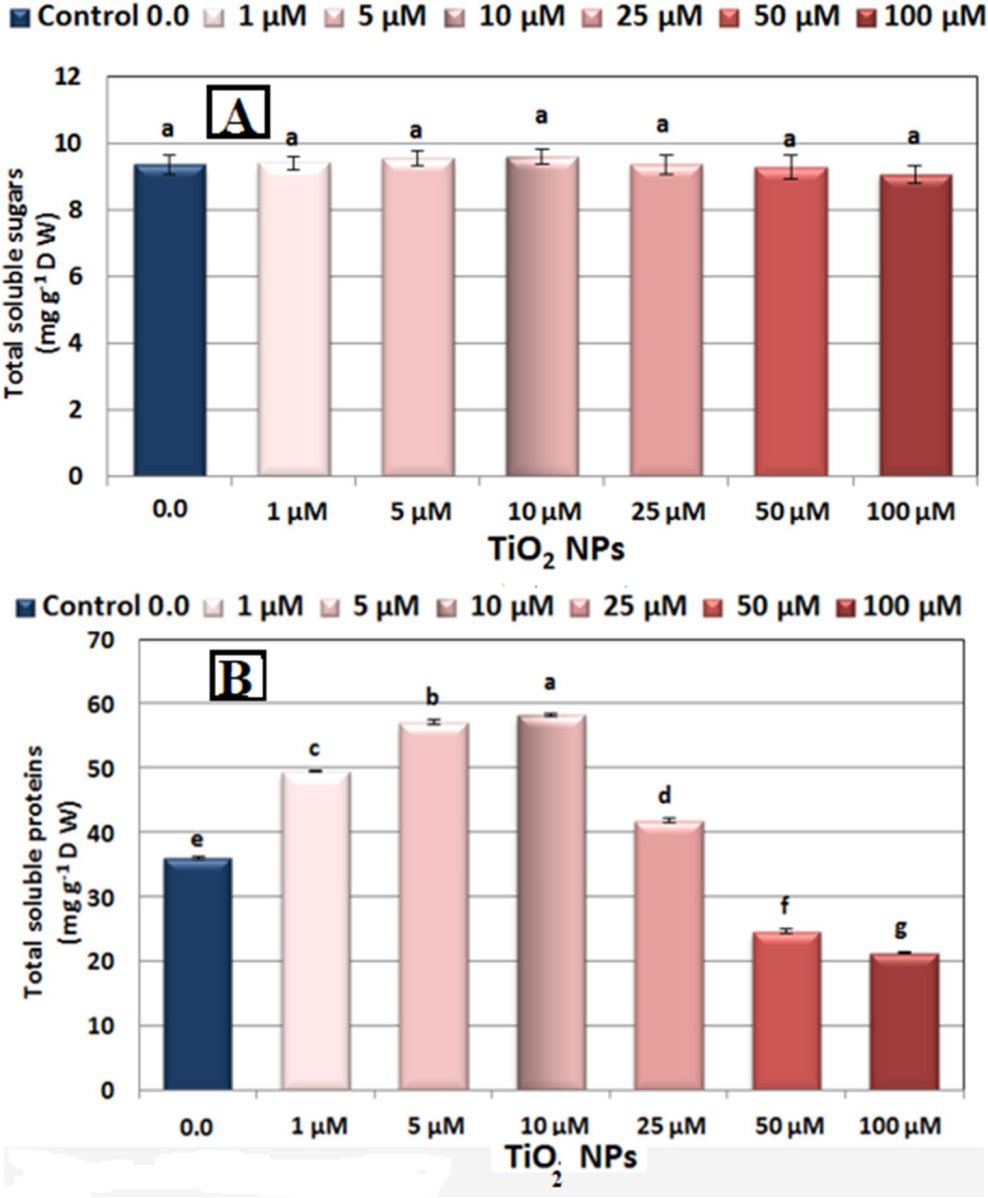
(**A**) Changes in soluble sugars, and (**B**) soluble proteins (mg g^− 1^ DW) in 7 day-old seedlings of Nubaria 4 variety of faba bean (*Vicia faba* L.) as affected by seed presoaking for 6 h in different concentrations of nano titanium (TiO_2_NPs ≈ 12.8 nm) solution at 0.0, 1, 5, 10, 25, 50 and 100 µM and grown at 25 °C ± 0.5. Each value represents the mean of 3 replicates. Different letters indicate significant differences between treatments (Duncan test *p* ≤ 0.05). Vertical bars represent ± SE.

The results presented in Fig. 4A, and 4B indicate that soaking faba bean seeds in TiO_2_NPs at concentrations of 1, 5, 10, and 25 µM significantly increased the levels of total soluble sugars (TSS) and total soluble proteins (TSP) in the seedlings. The maximum TSS (9.60 mg g^− 1^ DW) and TSP (58.22 mg g^− 1^ DW) levels were recorded when seeds were treated with a 10 µM TiO_2_NPs solution, compared to control values of 9.35 mg g^−1^ DW for TSS and 36.0 mg g^−1^ DW for TSP. However, as the TiO_2_NPs concentration surpassed 25 µM, there was a gradual decline in both TSS and TSP levels, with the lowest values of 9.07 mg g^−1^ DW for TSS and 21.33 mg g^−1^ DW for TSP observed at a concentration of 100 µM.

## Discussion

This research investigates the effects of varying concentrations of titanium dioxide nanoparticles (TiO_2_NPs; ranging from 1 to 100 µM) on the germination and growth of faba bean (*Vicia faba* L.) seeds. Specifically, it examines how these concentrations influence the germination percentage, seedling vigor index, and key growth parameters such as shoot and root lengths, along with fresh and dry weights of 7-day-old seedlings, in comparison to untreated seeds. Notably, the greatest germination performance was achieved when seeds were treated with 10 µM of TiO_2_NPs, contrasting with the control group. The enhancement in germination rates and seedling establishment is attributed to the seeds’ pre-treatment with lower concentrations of TiO_2_NPs, which facilitates increased water absorption and oxygen intake. Adequate water and oxygen availability within the imbibed faba bean seed likely accelerates enzymatic activities, including those of amylase and protease, thereby promoting the production of essential soluble sugars and amino acids necessary for early germination and seedling development. Additionally, this pre-treatment method softens the seed coat, facilitating the initial growth and emergence of the radicle, alongside associated biochemical processes^20^. Conversely, higher concentrations of TiO_2_NPs (50, 100 µM) negatively impacted germination efficiency, compared to the control. This adverse effect is hypothesized to stem from nanoparticle agglomeration at high concentrations, potentially inducing moisture stress and impairing water and oxygen uptake, thus hindering seed germination Raskar and Laware^21^. Such findings corroborate earlier reports suggesting that reduced germination could result from diminished water transport to the developing embryo and delayed breakdown of endosperm-bound biomolecules^22^.

These observations align with studies conducted on onion (*Allium cepa* L.) and vitex plants, where similar declines in germination percentages and radicle lengths were observed at elevated concentrations of TiO_2_NPs^23,24^.

The present investigation explores the effects of TiO_2_NPs at various concentrations on the seedling vigor index (SVI) of faba bean plants, as reflected through the germination percentage index. Notably, the application of 10 µM TiO_2_NPs led to the highest SVI values. These outcomes imply that employing TiO_2_NPs at concentrations not exceeding 10 µM as a pre-soaking treatment could serve as an efficient strategy to boost the growth of faba bean crops. Specifically, seed nano priming facilitated by TiO_2_NPs can enhance seed germination by organizing nanopores in the seed coat, thereby improving water uptake^25,26^. While NPs are primarily absorbed, they predominantly adhere to the seed surface as protective layers^27^. The presence of TiO_2_NPs triggers the generation of reactive oxygen species (ROS) within the seed, activates aquaporin genes, modifies starch-degrading enzymes, and alters seed tissue metabolism^26^. The accumulation of ROS upon NP uptake by the seed coat initiates a cascade reaction^28^. Localization of ROS is crucial for cellular communication within the endosperm and for the cleavage of glycosidic bonds between polysaccharides^29^. Superoxide dismutase (SOD) mediates the interaction between hydrogen peroxide (H_2_O_2_) and the phytohormone gibberellic acid (GA). GA, in turn, stimulates alpha-amylase to facilitate the hydrolysis of starch into easily soluble sugars, supporting embryonic development and, consequently, seed germination and seedling vigor^26,30^. These mechanisms are supported by the findings of Farahi et al.^24^, who found that titanium dioxide nanoparticles at 200 µgL^− 1^ exerted the most significant stimulation on germination metrics and seedling growth factors of vitex plantlets. However, as the concentration of TiO_2_NPs increased beyond 10 µM, the SVI began to diminish, with the most pronounced decreases observed at 50 and 100 µM. This decline could be linked to hormonal imbalances at higher TiO_2_NP concentrations, compared to control seedlings. Similarly, high concentrations of titanium oxide nanoparticles reduced SVI, compared to control vitex plantlets^24^.

This research examines the impact of titanium dioxide nanoparticles on the growth patterns of faba bean seedlings, revealing a consistent trend across various growth metrics. Treatments involving TiO_2_NPs up to a concentration of 25 µM significantly boosted the germination rate and subsequent growth parameters such as shoot and root lengths, as well as seedling fresh and dry weights. The beneficial effects at lower TiO_2_NPs concentrations may stem from de novo synthesis of new germination-enhancing substances like auxin and cytokinins, promotion of plant cell division, and the activation of certain hydrolytic enzymes (α-amylase, β-amylase, and protease) that optimize the use of seed reserves. Additionally, these treatments enhanced antioxidant activity and improved water absorption and utilization capabilities, akin to findings by Fujikura and Karssen^31^. Notably, the greatest improvements in shoot length, root length, seedling fresh, and dry weights were observed at a 10 µM concentration of TiO_2_NPs, Previous work by Mustafa et al.^32^ also highlighted the benefits of using TiO_2_NPs at 40 mg L^− 1^, showing significant enhancements in germination percentage, germination index, seedling vigor index, seedling length, and fresh weight in two wheat varieties. Conversely, when faba bean seeds were exposed to higher concentrations of TiO_2_NPs (50, 100 µM), a notable decline in growth characteristics was observed, which could be attributed to an increase in ROS levels due to oxidative stress. The metabolic activities within faba bean seeds are anticipated to escalate following exposure to TiO_2_NPs, leading to elevated concentrations of bioactive compounds. However, this excess may exceed the optimal thresholds needed for plant growth, causing stress and the production of growth inhibitory substances. This physiological imbalance is linked to heightened ROS activity, which are byproducts of mitochondrial respiration. These observations align with prior research indicating that external applications of high TiO_2_NP dosages adversely affect germination traits, and seedling development, whereas it has a favorable effect at low to moderate concentrations in both broad beans^33^, and wheat^34^.

Further, Li et al.^35^ utilized transmission electron microscopy (TEM) to reveal that high-dose TiO_2_NP treatments caused substantial damage to the outer layer of *Amaranthus mangostanus* seedlings, suggesting that ROS generated by high-dose TiO_2_NPs through photocatalysis can be detrimental and markedly hinder seedling growth.

The application of TiO_2_NPs at low concentration particularly at 1–10 µM significantly elevated the activity of key hydrolytic enzymes, namely α-amylase and protease, in faba bean (*Vicia faba*) seedlings compared to the untreated control. This enhancement is attributed to the upregulation of enzyme expression and the promotion of favorable physiological conditions within the seed during germination. Titanium nanoparticles may modulate signaling pathways that control the expression of hydrolytic enzymes like amylases, proteases, and lipases. By affecting gene expression, these nanoparticles may lead to an upregulation of enzyme production during germination, which in turn facilitates the rapid mobilization of stored reserves.

The elevated activity of α-amylase promotes the hydrolysis of starch into simpler sugars, thereby providing an immediate energy source for the developing seedling and contributing to faster and more synchronized germination. Likewise, the stimulation of protease activity enhances the breakdown of storage proteins, increasing the availability of amino acids necessary for seedling growth.

However, exposure to higher concentrations of TiO_2_NPs (100 µM) resulted in a marked decline in the activities of α-amylase and protease, likely due to inhibitory effects on enzyme synthesis at elevated nanopart icle levels. A comparable trend was reported in onion seedlings, where α-amylase and protease activities were enhanced at lower TiO_2_NP concentrations (10–40 µg mL^−1^) but diminished at higher levels (50 µg mL^−1^)^23^.

The increase in α-amylase activity observed during germination may be due to the de novo synthesis of the enzyme, as suggested by Filner and Varner^36^. Furthermore, Baron^37^ proposed that solutes generated from hydrolytic enzyme activity in the early stages of germination facilitate water uptake by contributing to the seed’s osmotic potential. Overall, enzymes such as amylases, proteases, and lipases play a pivotal role in degrading stored macromolecules during germination, thereby producing essential compounds required for seedling establishment. Specifically, αamylase and βamylase catalyze starch breakdown into sugars like maltose and glucose, which serve as primary energy sources for the developing embryo and emerging root–shoot axis^38^. Proteases, particularly cysteine-type enzymes, degrade storage proteins into free amino acids that are vital for new protein synthesis in the germinating seedling. Lipases likewise contribute by mobilizing lipid reserves, with their activity increasing significantly during germination to support energy demands^39^.

The enhancement of catalase, peroxidase, and superoxide dismutase activities at low-to-moderate concentrations of TiO_2_NPs suggests an induction of antioxidant defense mechanisms in response to mild oxidative stress. This is consistent with previous reports indicating that low levels of nanoparticles can stimulate ROS production, which in turn activates antioxidant enzymes to maintain cellular redox balance^40^. Catalase and peroxidase are key enzymes responsible for scavenging hydrogen peroxide (H_2_O_2_), while SOD catalyzes the dismutation of superoxide radicals into H_2_O_2_ and oxygen^41^. POX, and CAT play significant roles in the detoxification and decomposition of hydrogen peroxide within cells^42^. Their increased activities at 1–25 µM may reflect the plant’s adaptive response to mitigate nanoparticle-induced oxidative stress and maintain cellular homeostasis^43,44^. However, the decline in enzyme activities at higher concentrations (50–100 µM) suggests that excessive TiO_2_NP accumulation may overwhelm the antioxidant system, leading to enzyme inhibition or cellular damage. High doses of nanoparticles have been reported to disrupt metabolic activities, denature proteins, and impair enzymatic function^23,45^. This biphasic response highlights the importance of dosage in determining the physiological effects of nanomaterials on plants.

The results of this study indicate that soaking faba bean (*Vicia faba*) seeds in low to moderate concentrations of TiO_2_NPs, particularly within the range of 1 to 25 µM, positively influences seedling biochemistry by enhancing both total soluble sugars and total soluble proteins. This enhancement is likely linked to the stimulation of hydrolytic enzymes, such as amylases and proteases, which are essential for breaking down complex macromolecules like starch, proteins, and lipids into simpler, metabolically available forms. Such enzymatic activity not only improves metabolic efficiency but may also upregulate genes involved in protein synthesis, ultimately contributing to increased protein accumulation in seedlings. These physiological improvements translate into enhanced seedling vigor, faster growth rates, and better overall health.

Our findings are supported by earlier studies. For instance, Wang et al.^46^ demonstrated that amylase activity progressively increases during the early stages of germination, facilitating the conversion of starch reserves into soluble sugars necessary for embryonic development. Marambe et al.^47^ further established a strong correlation between α-amylase activities, seed water uptake, and subsequent seed germination percentage, alongside a linear relationship between amylase activity and starch degradation, leading to an increase in sugar content in treated sorghum seeds.

Protease activity also plays a crucial role, as shown by Ikuko and Hiroshi^48^, who reported that proteolytic enzymes contribute to the complete degradation of storage proteins, releasing free amino acids required for de novo protein synthesis in the embryonic axis. This supports the observed increase in soluble protein content in our study.

The mechanisms underlying these enhancements are consistent with the findings of Mahakham et al.^25^, who reported that nano-priming promotes germination through various pathways, including the creation of nanopores for enhanced water uptake, upregulation of aquaporin genes, rebalancing of ROS and antioxidant systems in seeds, generation of hydroxyl radicals for cell wall loosening, and the catalyst acceleration of starch hydrolysis, compared to unprimed control treatments.

Additional studies lend further support to the observed trends. For instance, Abdel Latef & Hornung^49^ reported that the application of 0.01% TiO_2_ nanoparticles significantly enhanced soluble sugar content in green bean (*Phaseolus vulgaris*) plants. Similarly, Seyed et al.^50^ observed a comparable increase in soluble sugar accumulation in *Vitex* species following treatment with 200 ppm TiO_2_ NPs. In addition, foliar application of TiO_2_ NPs was found to markedly elevate protein content in mung bean (*Vigna radiata*) as demonstrated by Raliya et al.^9^. These findings collectively highlight the positive role of TiO_2_NPs in enhancing key biochemical constituents across various plant species.

However, it is important to note that higher TiO_2_NP concentrations (exceeding 25 µM) had detrimental effects on these biochemical parameters. This decline is likely due to oxidative stress and disruptions in metabolic activity caused by excessive nanoparticle exposure, a pattern consistent with previous reports of nanoparticle-induced phytotoxicity.

## Conclusions

The present study concludes that soaking faba bean seeds in titanium dioxide nanoparticle solutions at concentrations up to 25 µM enhances the seeds’ ability to efficiently mobilize stored reserves. This improvement is achieved by increasing the activity of hydrolytic (α-amylase, and protease), and antioxidant enzymes along with elevated levels of total soluble sugars and proteins, which in turn leads to faster germination, more uniform seedling growth, and increased vigor during early plant development. However, the effect of TiO_2_ nanoparticles is highly concentration-dependent. While moderate concentrations stimulate enzyme activity, higher concentrations (100 µM) can induce toxicity, inhibit enzyme function and significantly reduce both germination and seedling growth compared to the control. These findings emphasize the importance of optimizing TiO_2_ nanoparticle concentrations for safe and effective use in agricultural applications.

## Data Availability

Authors can confirm that all relevant data are included in the given article.
